# Reproducibility of abnormality detection on chest radiographs using convolutional neural network in paired radiographs obtained within a short-term interval

**DOI:** 10.1038/s41598-020-74626-4

**Published:** 2020-10-15

**Authors:** Yongwon Cho, Young-Gon Kim, Sang Min Lee, Joon Beom Seo, Namkug Kim

**Affiliations:** 1 Department of Biomedical Engineering, Asan Medical Institute of Convergence Science and Technology, Asan Medical Center, University of Ulsan College of Medicine, Seoul, Republic of Korea; 2 Department of Convergence Medicine, University of Ulsan College of Medicine, Asan Medical Center, Seoul, South Korea; 3 Department of Radiology and Research Institute of Radiology, University of Ulsan College of Medicine, Asan Medical Center, Seoul, South Korea

**Keywords:** Computational biology and bioinformatics, Biological techniques, Software, Biomarkers, Diagnostic markers, Diagnosis, Medical imaging, Medical research, Experimental models of disease, Mathematics and computing, Computer science, Engineering, Biomedical engineering

## Abstract

We evaluated the reproducibility of computer-aided detections (CADs) with a convolutional neural network (CNN) on chest radiographs (CXRs) of abnormal pulmonary patterns in patients, acquired within a short-term interval. Anonymized CXRs (n = 9792) obtained from 2010 to 2016 and comprising five types of disease patterns, including the nodule (N), consolidation (C), interstitial opacity (IO), pleural effusion (PLE), and pneumothorax (PN), were included. The number of normal and abnormal CXRs was 6068 and 3724, respectively. The number of CXRs (region of interests, ROIs) of N, C, IO, PLE, and PN was 944 (1092), 550 (721), 280 (538), 1361 (1661), and 589 (622), respectively. CXRs were randomly allocated to training, tuning, and test sets in 70:10:20 ratios. Two thoracic radiologists labeled and delineated the ROIs of each disease pattern. The CAD system was developed using eDenseYOLO. For the reproducibility evaluation of developed CAD, paired CXRs of various diseases (N = 121, C = 28, IO = 12, PLE = 67, and PN = 20), acquired within a short-term interval from the test sets without any changes confirmed by thoracic radiologists, were used to evaluate CAD reproducibility. Percent positive agreement (PPAs) and Chamberlain’s percent positive agreement (CPPAs) were used to evaluate CAD reproducibility. The figure of merit (FOM) of five classes based on eDenseYOLO showed N-0.72 (0.68–0.75), C-0.41 (0.33–0.43), IO-0.97 (0.96–0.98), PLE-0.94 (0.92–95), and PN-0.87 (0.76–0.93). The PPAs of the five disease patterns including N, C, IO, PLE, and PN were 83.39%, 74.14%, 95.12%, 96.84%, and 84.58%, respectively, whereas the values of CPPAs were 71.70%, 59.13%, 91.16%, 93.91%, and 74.17%, respectively. The reproducibility of abnormal pulmonary patterns from CXRs, based on deep learning-based CAD, showed different results; this is important for assessing the reproducible performance of CAD in clinical settings.

## Introduction

Chest radiographs (CXRs) are the first diagnostic imaging parameters for screening patients with non-specific thoracic symptoms in general clinical practice. Many CXR-based studies have been conducted for thoracic diseases due to their easy availability, efficiency, and low cost. However, instances of missed diagnosis of diseases on CXRs are common in retrospective examinations, even if the initial diagnosis was made by experienced radiologists, due to the practical burden on radiologists associated with the examinations of all CXRs while maintaining high diagnostic quality^1–3^. Computer-aided detection (CAD) system has shown promise in detecting potentially abnormal pulmonary patterns on CXRs^4–6^. CAD could be used to assist the identification of pulmonary lesions on CXRs by lesion demarcation and attention maps. Hoop et al.^7^ reported that CAD did not significantly improve cancer diagnostic performance on CXRs as the examiner was unable to effectively distinguish between the true-positive and false-positive marks. A recent study on a large number of CXRs^8^ reported the diagnostic sensitivity of stand-alone CAD to be 71%, with 1.3 false-positive results per image. Although the performance of CAD has improved significantly, better sensitivity and low false-positive rates are required for its integration into clinical use. Another important aspect of concern for using CAD on CXRs is its reproducibility.

Recently, the use of multiple CAD systems has been implemented with the picture archiving and communication system (PACS)^9–14^. This seamless integration of CAD and PACS has vastly improved the efficiency of routine clinical practice, reducing the average image reading time, and increasing reader sensitivity^11^. Diagnostic systems have been developed that successfully integrate deep learning with a convolutional neural network (CNN) and CAD, to assess CXRs in cases of multiple lesions. Lakhani et al.^9^ demonstrated accurate diagnosis of tuberculosis from CXRs using deep learning, with an area under the receiver operating characteristic curve (AUC) of 0.99 that surpassed an AUC of 0.87–0.90 reported by a previous study using support vector machines^10^. Similarly, Islam^11^ reported on the diagnosis of pulmonary abnormalities on CXRs and found that the ensemble method with deep learning provided the highest accuracy for detecting abnormalities. These previous studies did not address the reproducibility of CAD in CXRs of same patients within a short-term interval; they reported on the changes in CXRs^15^. We previously reported that the reproducibility of the CAD system could be one of the important indicators for the performance by the four different algorithms, using only the nodule, on CXRs^16^. In this study, we evaluated the reproducibility of CAD of multiple lesions on CXRs using paired images acquired within a short-term interval from the test sets and in those where no changes were reported by expert thoracic radiologists of our institution.

## Materials and methods

**Figure 1:**
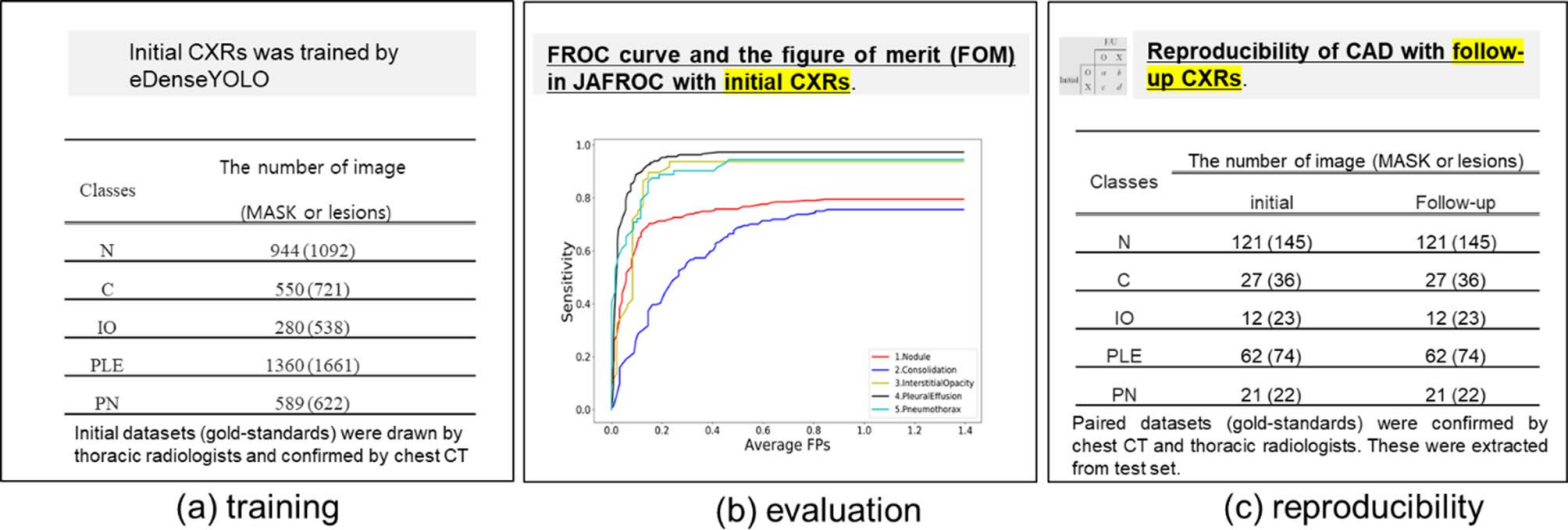
The workflow of the reproducibility of CAD based on deep learning. (**a**) Training with initial CXRs using eDensYOLO, (**b**) evaluation of (**a**), and (**c**) reproducibility of CAD with follow-up CXRs (PPA and CPPA).

Our institutional review board approved this retrospective cohort study and the requirement for informed consent was waived. Figure 1 outlines the study workflow.

### Datasets

**Figure 2:**
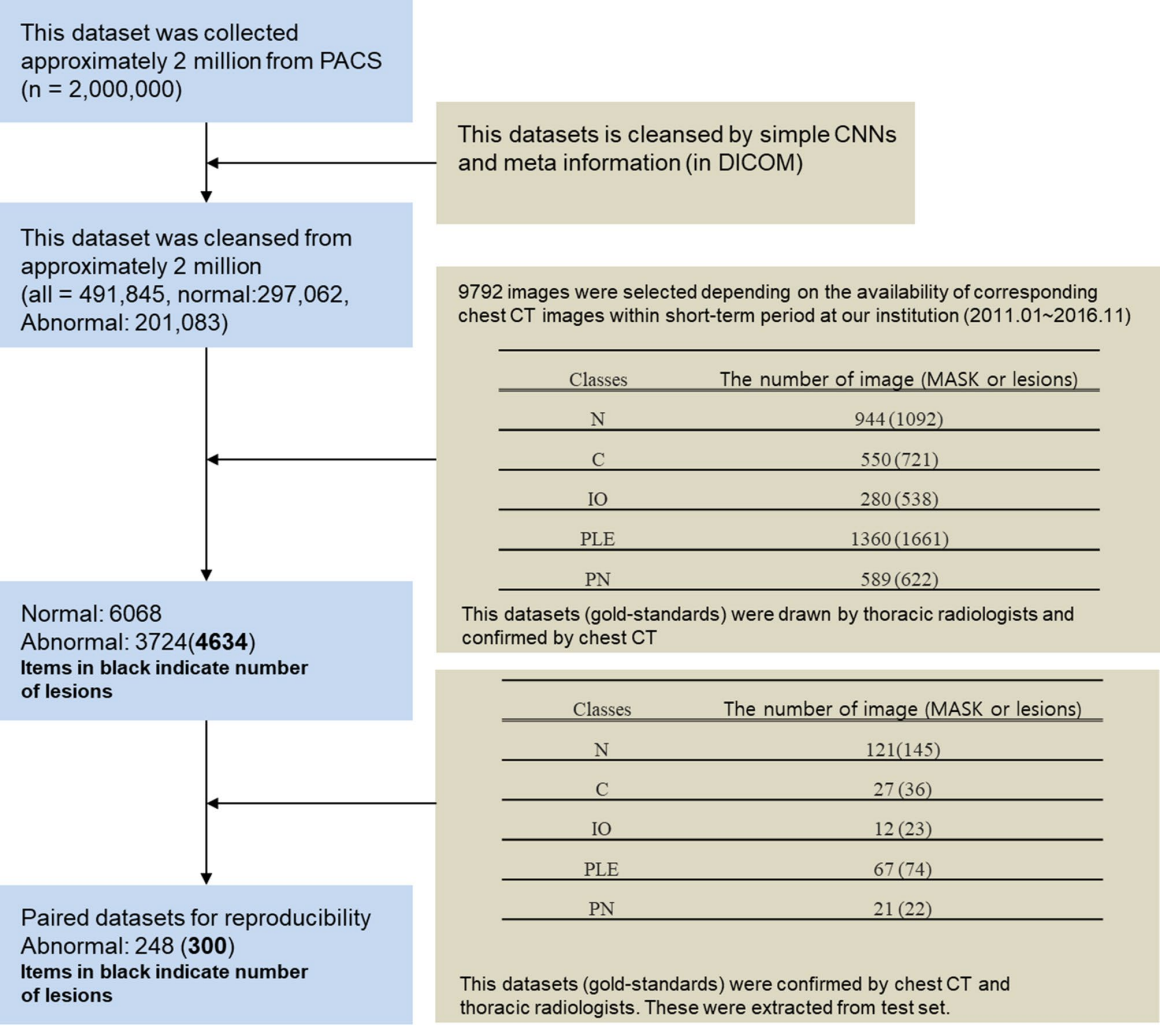
Dataset-trees used in our study from AMC. *AMC* Asan Medical Center.

**Figure 3:**
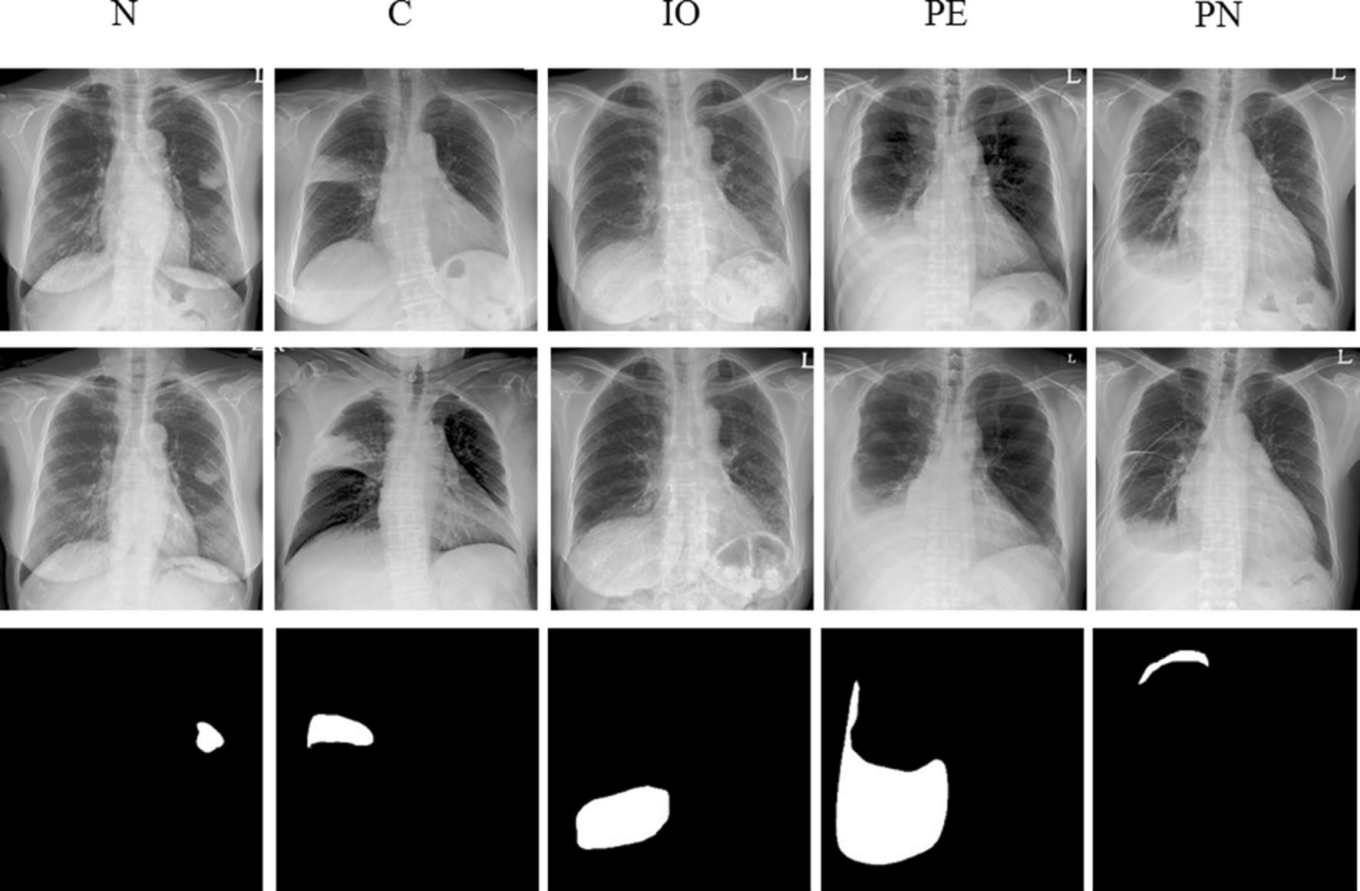
Representative examples of initial chest radiographs (CXRs) taken from each patient within a short-term interval (up rows), follow-up images of CXRs taken from each patient within a short-term interval (middle rows), and gold-standards labeled by two thoracic radiologists (bottom rows). *N* nodule, *C* consolidation, *IO* interstitial opacity, *PLE* pleural effusion, *PN* pneumothorax in Fig. 2.

All CXRs scanned by the computed radiography (CR) or digital radiography (DR) system were downloaded from PACS at ASAN Medical Center. From the 491,845 CXRs images, 9792 images were selected depending on the availability of corresponding chest CT images from January 2011 to November 2016. This dataset was derived from approximately 2 million digital imaging and communications in medicine images from the initial examination of normal and abnormal CXRs identified by hospital diagnostic code. Raw datasets extracted PACS system included the bone, MRI, skull, and others. We cleansed these types for CXRs. Generating a strong label during a short period in a hospital is difficult. We decided on the optimal number of datasets for training and then drew it directly for the radiologist. We developed in-house software for delineating the ROIs as a reference mask for the diseased lesions so that the in-house radiologists could efficiently draw the ROIs on the lesions with existing software over many CXRs. This software included shortcuts for mapping and loading of the next image and a user interface for auto-loading the images, moving an image to the trash, auto-saving, and annotating an ROI. Two thoracic radiologists, with five and ten years of experience, shared their opinions before labeling the diseased lesions. Normal and abnormal datasets with nodules (N including mass)/consolidation (C) or interstitial opacity (IO) were confirmed by chest CT. For pleural effusion (PLE) and pneumothorax (PN), detected via CXRs, confirmation was reached through consensus of two thoracic radiologists with corresponding chest CT images due to the difficulty of detecting PLE and PN in CXRs. Thereafter, two thoracic radiologists delineated the exact boundaries of the lesions using our software. If co-located lesions were observed in the CXRs, the separated boundaries of overlapped shapes in CXRs were drawn by thoracic radiologists. The study design’s drawing of the ROIs for one of the five disease patterns of lesions, by simultaneously referring to the patients’ paired computed tomography images, is shown in Fig. 2. The number of normal and abnormal CXRs was 6068 and 3724, respectively. The number of CXRs with N, C, IO, PLE, and PN patterns was 944 (1092), 550 (721), 280 (538), 1361 (1661), and 589 (622), respectively. The bracket refers to the number of references drawn by two thoracic radiologists. To detect the five disease patterns, all CXRs were randomly split into training, tuning, and test sets in 70:10:20 ratios as for final CAD assessment with initial CXRs (Table 1). To evaluate the reproducibility of CAD, follow-up CXRs within one week with little change of lesions were selected in the entire follow-up CXRs. Table 2 shows the paired datasets for the initial and follow-up CXRs extracted from the test datasets of Table 1, which were confirmed by expert thoracic radiologists. The average intervals between the initial and follow-up CXRs were: N (2.09 ± 1.33), C (1.33 ± 1.15), IO (2.33 ± 2.21), PLE (1.63 ± 1.69), and PN (1.67 ± 2.18). Despite the lack of change according to the pattern of the lesion between the paired initial and follow-up CXRs, these datasets may differ in their image quality, angle, and position (Fig. 3).

**Table 1:** The number of chest radiograph (CXR) datasets for each disease pattern in the training and test sets extracted from our institution.

Classes	Number of images in training set (with tuning set)	Number of images in test set
N	756	188
C	426	124
IO	232	48
PLE	1113	248
PN	520	69
Total	3095	677

Nodule (N), consolidation (C), interstitial opacity (IO), pleural effusion (PLE), and pneumothorax (PN).

**Table 2:** The number of paired chest radiograph (CXR) datasets for the initial and follow-up CXR images including the five disease patterns.

Classes	Pair datasets for evaluating reproducibility
N	121
C	28
IO	12
PLE	67
PN	20
Total	248

Nodule (N), consolidation (C), interstitial opacity (IO), pleural effusion (PLE), and pneumothorax (PN).

### Methods

**Figure 4:**
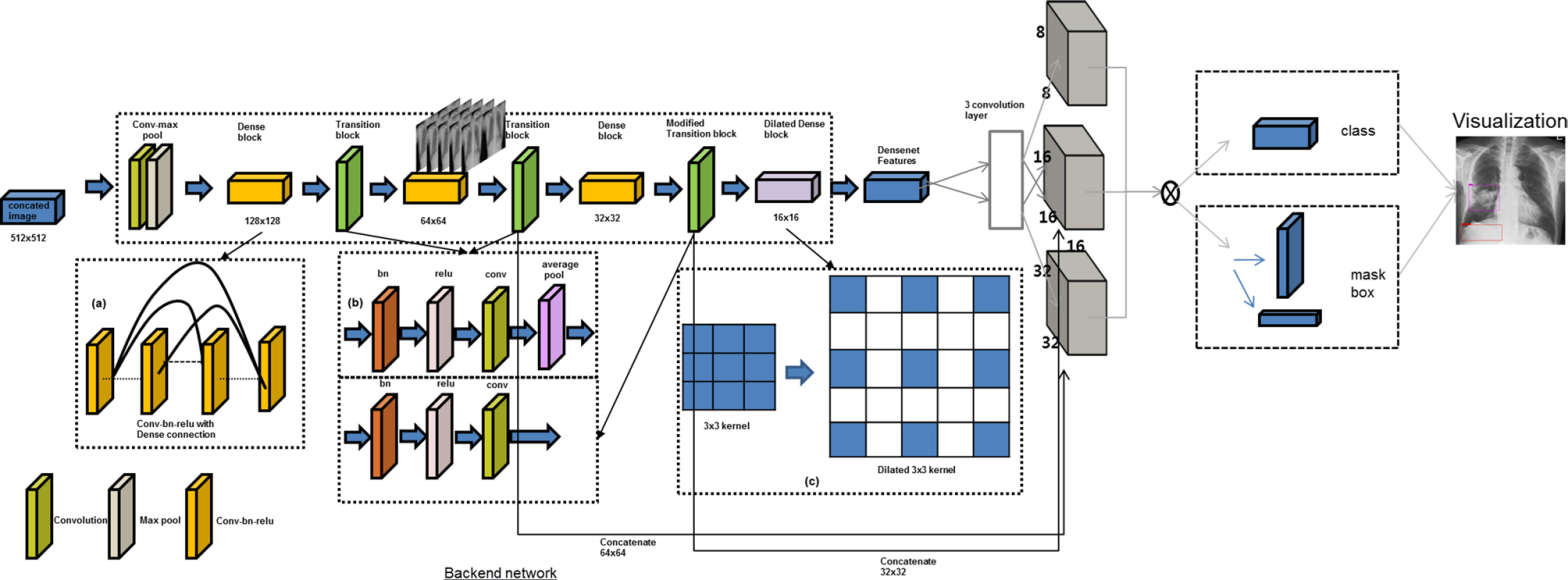
Architecture of the eDenseYOLO of which backend network is DenseNet201. The output layers of eDenseYOLO, which is You Only Look Once v2 with DenseNet201, were modified for improved robustness to the variable size of disease patterns. If the input resolution was 256 × 256, the feature map for the last layer was 8 × 8, 16 × 16, and 32 × 32 with skip connection.

We used the eDenseYOLO system, with modifications from its original architecture of you only look once (YOLO) v2^17^, with a multi-scale scheme to improve the performance of CAD (Fig. 4). This network is deeply fine-tuned and trained with the ROIs of disease patterns.

We fine-tuned the whole network using pre-trained weights of ImageNet training. After training, we examined the location and classification of various disease patterns among the test sets of CXRs. The main use of YOLO v2^17^ was to divide each image by S × S grid, with direct regression to find the B bounding boxes, confidence for those boxes, and the probabilities of each class within each grid cell. Each grid cell has conditional class probabilities of each class for all the bounding boxes^17^. The loss function of YOLO v2 includes both bounding boxes and object classification as follows:

1 $$ ss = \lambda_{coord} \mathop \sum \limits_{i = 0}^{{S^{2} }} \mathop \sum \limits_{j = 0}^{B} 1_{ij}^{obj} \left( {x_{i} - \hat{x}_{i} } \right)^{2} + \left( {y_{i} - \hat{y}_{i} } \right)^{1} $$

2 $$ + \lambda_{coord} \mathop \sum \limits_{i = 0}^{{S^{2} }} \mathop \sum \limits_{j = 0}^{B} 1_{ij}^{obj} \left[ {\left( {\sqrt {w_{i} } - \sqrt {\hat{w}_{i} } } \right)^{2} + \left( {\sqrt {h_{i} } - \sqrt {\hat{h}_{i } } } \right)^{2} } \right] $$

3 $$ + \mathop \sum \limits_{i = 0}^{{S^{2} }} \mathop \sum \limits_{j = 0}^{B} 1_{ij}^{obj} \left( {C_{i} - \widehat{{C_{j} }}} \right)^{2} $$

4 $$ + \lambda_{noobj} \mathop \sum \limits_{i = 0}^{{S^{2} }} \mathop \sum \limits_{j = 0}^{B} 1_{ij}^{noobj} \left( {C_{i} - \widehat{{C_{j} }}} \right)^{2} $$

5 $$ + \mathop \sum \limits_{i = 0}^{{S^{2} }} 1_{i}^{obj} \mathop \sum \limits_{c \in classed} (p_{i} \left( c \right) - \hat{p}_{i} \left( C \right))^{2} $$

where [(1), (2)] are five elements wherein the losses of the bounding box are the coordinates [(3), (4)] for the confidence score of object[s] or no object in the grid, and (5) the class probability for *ith* grid cell and *jth* box. The equation calculates the losses of *x, y, w,* and h to predict the bounding box *j* of grid cell *i* where the object exists. It takes the square root to reflect the small deviation from large boxes and then calculates the error sum of squares (SSE). Even with the same error, the larger box has a lower impact on the intersection of union (IOU). To predict the bounding box *j* of grid cell *i* where the object does or does not exist, it calculates the loss to the confidence score (5). Finally, it calculates the loss of conditional class probability for grid cell *i* where the object exists. The *λ_coord* is a parameter for balancing loss and other losses with the coordinates (*x, y, w, h*). The λ_noobj is a parameter for balancing between boxes with or without objects. The sum of these loss functions is updated and used to infer lesions in the image. The detection result must be 0.5 or more to reflect the importance of classification and detection^17^.

In Fig. 4, the output layers of YOLO v2 with DenseNet201 as eDenseYOLO are shown and modified to be robust to the variable sizes of various disease patterns. We fine-tuned with the whole network using the pre-trained weight. For example, if the input was 256 × 256, the feature map for the last layer was 8 × 8. Next, we up-sampled the last layer by 2 × and merged our up-sampled features with the forward skip connection using concatenation in Fig. 4. Therefore, the last layer includes multi-scale feature maps (8 × 8, 16 × 16, 32 × 32, …, N × N). A total of 5 arbitrary anchor boxes per each feature map were used to forward and backpropagation in CXRs. This concept maintains various predictions for specific disease patterns in CXRs. Therefore, this network predicts the class confidence scores and locations of its bounding box to detect multiple lesions in the CXRs.

For training and inference, all the CXRs were resized into 1000 by 1000 pixels with bi-linear interpolation due to the lack of current GPU memory, which was the optimal image matrix size for detecting nodules or small size lesions^18^. For enhancing the performance of the CAD model, the training datasets were pre-processed using histogram matching to match the histogram distributions of all images. We used image augmentation techniques, including brightness, contrast, Gaussian noise, blur, inversion, sharpness, and geometric augmentation—including shift, zoom, and rotation. These augmentations helped alleviate scanner-specific biases and were used to improve the robustness of the neural networks against additional sources of variability, that were unrelated to the radiological classes. These datasets were loaded on a Graphics Processing Unit (GPU) devbox server with *Ubuntu* 14.04, CUDA 8.0, and *cuDNN* 5.1 (NVIDIA Corporation), part of the NVIDIA deep learning software development kit, on a DARKNET platform (C++ version)^17^. The GPU server contained four 22 GB P40. We used an initial learning rate of 0.001 that decayed by a factor of 10, each time the tuning validation loss plateaued after an epoch and chose the model with the lowest tuning loss with *ADAM* optimizer.

### Evaluation metrics of reproducibility

**Figure 5:**
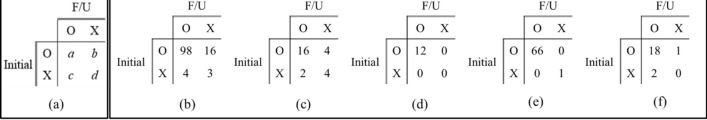
(**a**) An example of confusion matrix for reproducibility of CAD between initial and follow-up CXRs at cut-off threshold (0.6). Reproducibility result matrices of eDenseYOLO for initial and follow-up CXRs by (**b**) N, (**c**) C, (**d**) IO, (**e**) PLE, and (**f**) PN. *Note*: O: correct prediction, X: wrong prediction.

To analyze the reproducibility of CAD for diagnosing the five disease patterns, including N, C, IO, PLE, and PN, we selected PPA (6)^19,20^ and CPPA (6)^19,20^. This evaluation metrics are commonly used for reproducibility or evaluating the agreement of two tests.

6 $$ {\text{percent positive agreement }}\left( {{\text{PPA}}} \right) = 100 \times \frac{2a}{{2a + b + c}} $$

7 $$ {\text{chamberlain's percent positive agreement }}\left( {{\text{CPPA}}} \right) = 100 \times \frac{a}{a + b + c} $$

where a is the number of cases in which the lesions on the initial and follow-up CXRs were equally detected, and b and c are the numbers of cases in which the lesions were only detected in the initial or follow-up CXRs. D is the number of cases in which the lesions were not equally detected in both the initial and follow-up CXRs. D was not used for calculating PPA and CPPA because we wanted to determine how to consistently measure CAD based on the deep learning prediction of lesions (N, C, IO, PLE, and PN) in follow-up CXRs. The 'd' was not used for measurements such as PPA or CPPA as we wanted to measure how consistently the deep learning model predicted lesions for patients with diseases such as nodules or masses in follow-up CXR. Figure 5a shows an example of a confusion matrix to measure PPA and CPPA. Statistical analyses were used to evaluate a pair of agreement measures for reproducibility.

### Ethical approval

Experiments on humans and/or the use of human tissue samples have not been conducted in this study. In addition, no organs/tissues were procured from prisoners in this study. We confirm that all experiments were performed following relevant guidelines and regulations.

## Results

**Figure 6:**
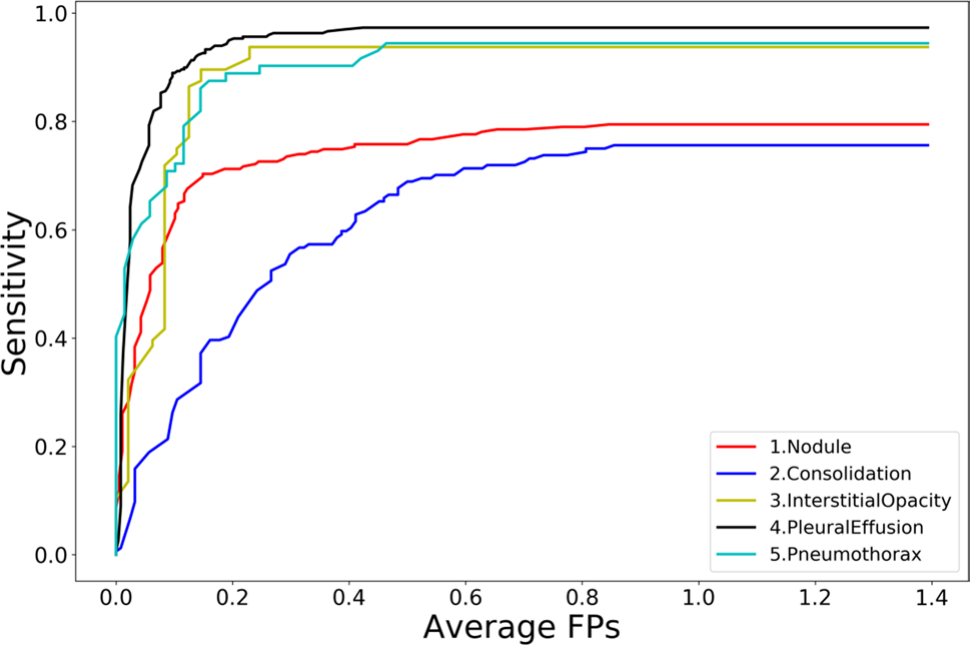
Free-response receiver operating characteristic of the computer-aided detection on five disease patterns with eDenseYOLO.

We first evaluated the performance of the algorithm of eDenseYOLO using a free-response receiver operating characteristic (FROC) curve in Fig. 6. To measure accuracy between the predicted bounding box and labels of ground truth, we used the IOU and confidence score (classification value of five lesions). When IOU was over 0.5, the predicted lesions in CXRs were regarded as correct. An FROC curve according to each confidence score was evaluated. To evaluate the reproducibility of CAD based on deep learning, the cut-off threshold (0.6) was determined using sensitivity and average false positives in eDenseYOLO. These cut-off thresholds for reproducibility were determined empirically as the number of average false positives 0.1, 0.2, 0.3, 0.4, 0.5, and 0.6 in the FROC curve of the validation set in Figs. 1 and 2. In this cut-off threshold (0.6), the CAD recall in the test set (initial CXRs) including N, C, IO, PLE, and PN in Table 1 was 78%, 71%, 93%, 97%, and 88%. Paired datasets of various diseases, acquired within a short-term interval from the procurement of the test in CXRs were, used to validate the reproducibility of our CAD (Table 2) at this cut-off threshold (0.6). Thereafter, we calculated the confusion matrix for the initial and follow-up CXRs for the reproducibility of CAD (Fig. 5). This statistical analysis is different from the FROC curve and the method to evaluate the reproducibility between initial and follow up CXRs. The PPAs and CPPAs for N, C, IO, PLE, and PN were 90.74%, 84.21%, 100%, 100%, 92.31% and 83.05%, 72.73%, 100%, 100%, and 85.71%, respectively. The average values of the PPAs and CPPAs were 93.45% and 88.30%, respectively.

Table 3 shows the figure of merit (FOM) of JARFOC and reproducibility comparisons in terms of PPAs and CPPAs. The PPAs were evaluated at 83.39 ± 4.12%, 74.14 ± 5.13%, 95.12 ± 5.78%, 96.84 ± 1.62%, and 84.58 ± 9.99%, respectively. The CPPAs were measured at 71.70 ± 6.29%, 59.13 ± 6.88%, 91.16 ± 8.84%, 93.91 ± 3.11%, and 74.17 ± 14.55%, respectively. PLE showed the highest PPA of 96.84 ± 1.62% and CPPA of 93.91 ± 3.11%.

**Table 3:** Figure of merit (FOM) (95% confidence interval) of jackknife free-response receiver operating curve (JAFROC) and reproducibility analysis of PPA and CPPA of CAD-based detection algorithms for five classes.

Classes	FOM (95% CI)	PPA (%)	CPPA (%)
N	0.72 [0.68–0.75]	83.39 ± 4.12	71.70 ± 6.29
C	0.41 [0.33–0.43]	74.14 ± 5.13	59.13 ± 6.88
IO	0.97 [0.96–0.98]	95.12 ± 5.78	91.16 ± 8.84
PLE	0.94 [0.92–0.95]	96.84 ± 1.62	93.91 ± 3.11
PN	0.87 [0.76–0.93]	84.58 ± 9.99	74.17 ± 14.55

*FOM* Figure of Merit.

**Figure 7:**
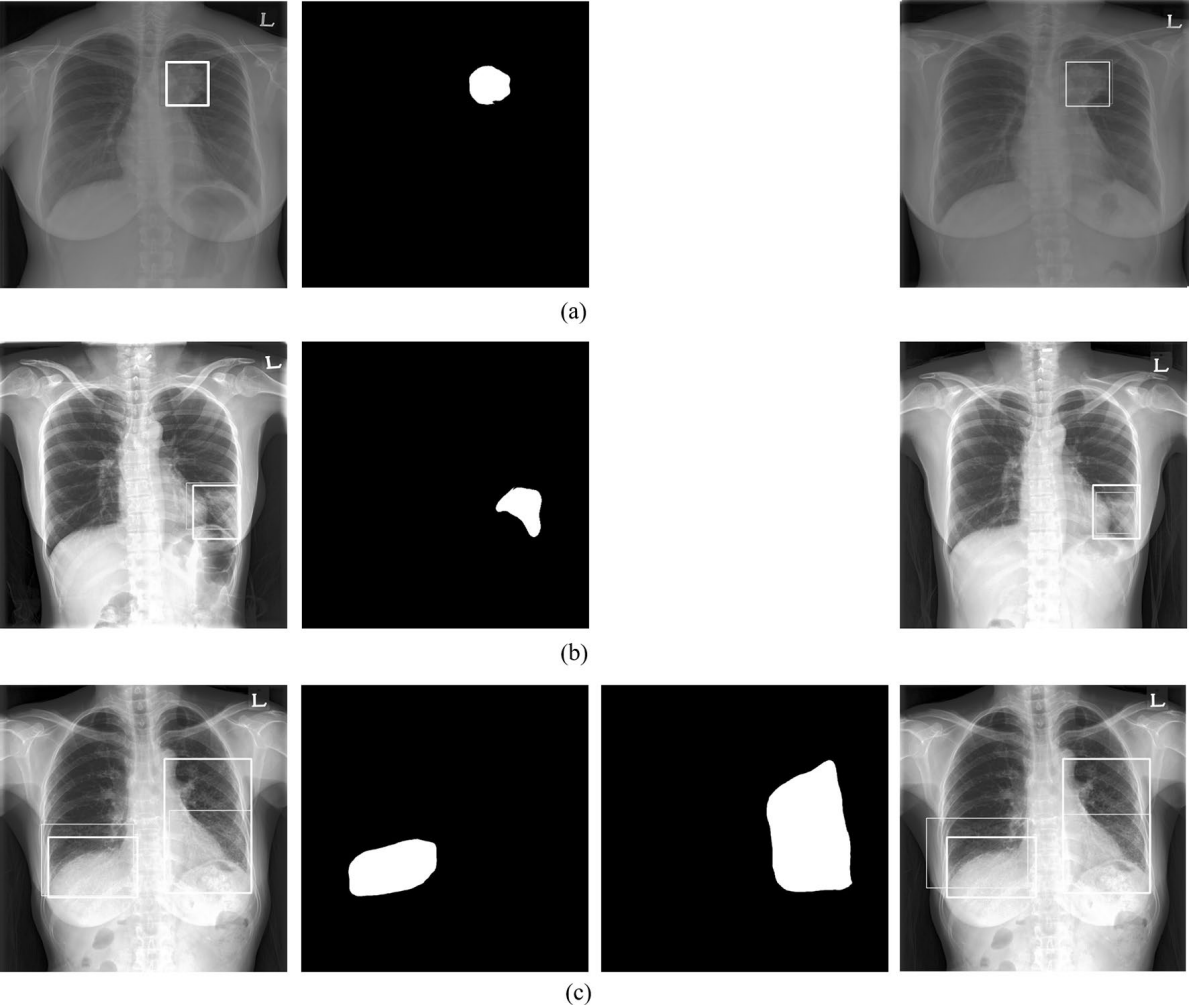
Examples of reproducibility between initial CXRs and follow-up CXRs; the inference of initial CXRs (left column), gold-standards labeled by two thoracic radiologists (the two columns in middle), and the inference of follow-up CXRs (right column). (**a**) positive agreement of N, (**b**) positive agreement of C, and (**c**) positive agreement of PLE on the initial and follow-up CXRs. Colors represent the disease types (N, red; C, green; IO, yellow; PLE, blue; PN, pink; and gold-standard, white).

**Figure 8:**
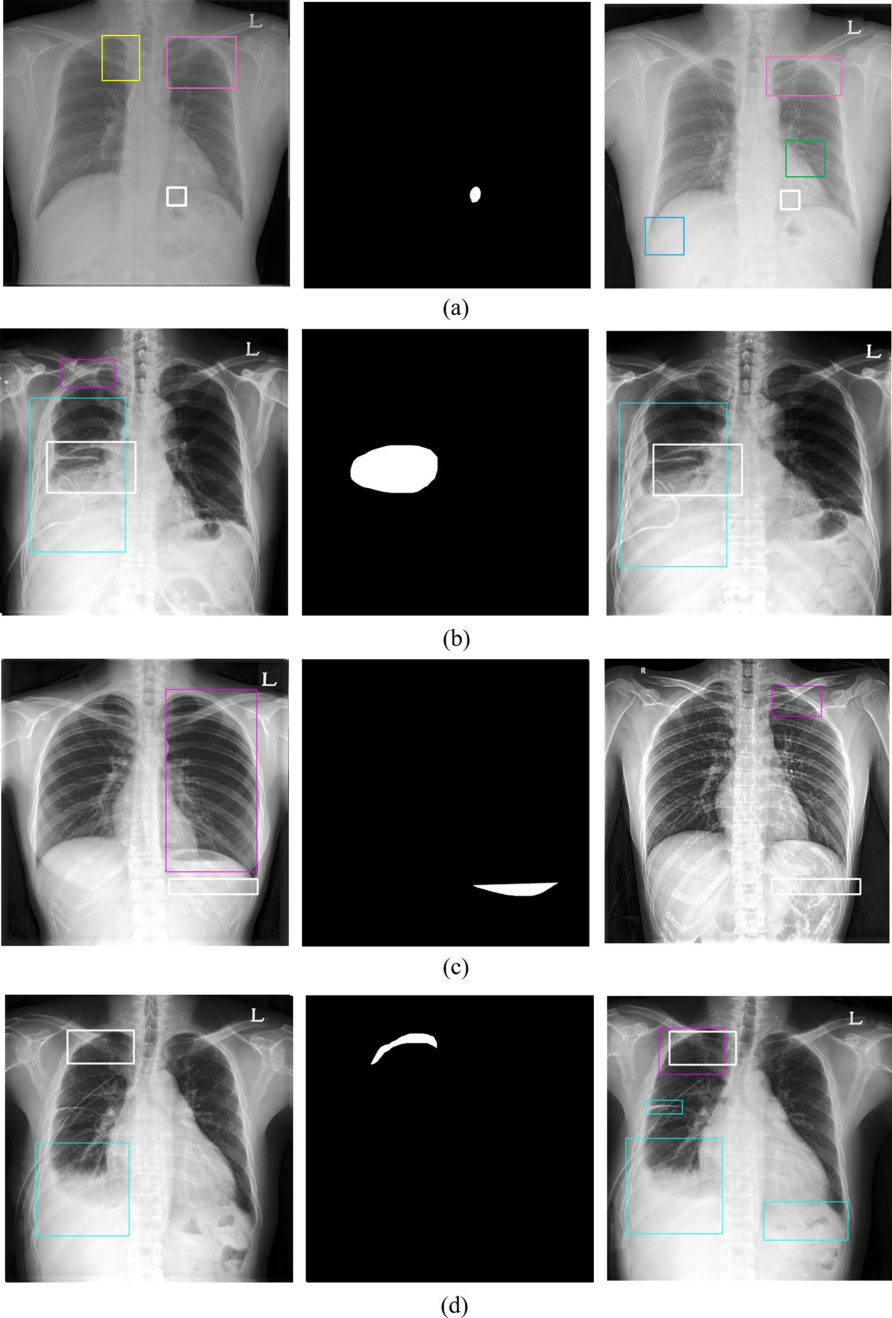
Examples of reproducibility between initial CXRs and follow-up CXRs; the inference of initial CXRs (left column), gold-standards labeled by two thoracic radiologists (the one column in middle), and the inference of follow-up CXRs (right column). (**a**) Negative agreement of N, (**b**) negative agreement of C, and (**c**) negative agreement of PLE, and (**d**) negative agreement of PN on the initial and follow-up CXRs. The colored boxes of each image represent false positives (N, red; C, green; IO, yellow; PLE, blue; PN pink; and gold-standard, white).

**Figure 9:**
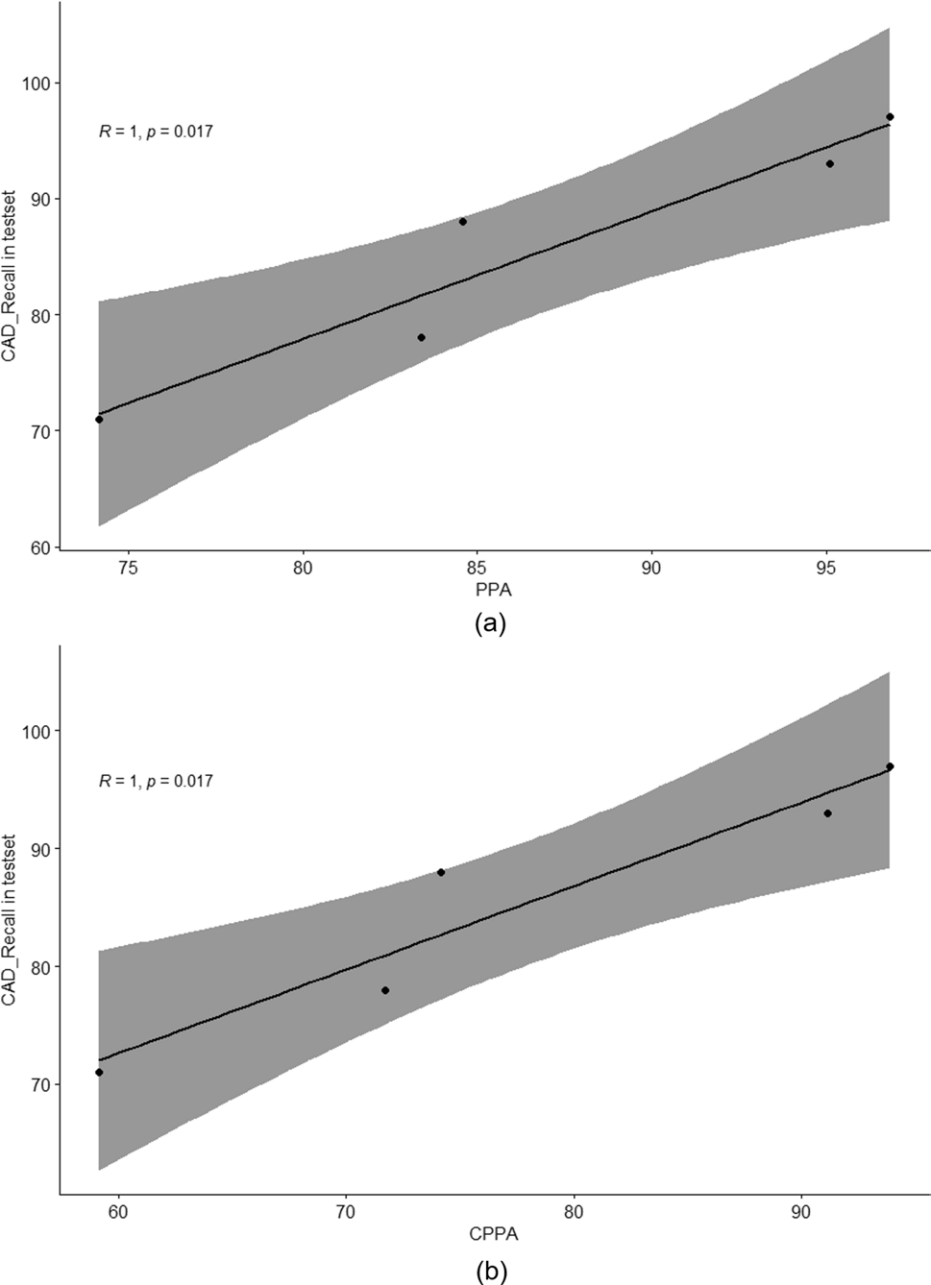
The plots with Spearman's rank correlation. (**a**) The recall of CAD in the test set and PPA, and (**b**) the recall of CAD in test set and CPPA.

The consolidation disease type demonstrated the worst reproducibility whereas the interstitial opacity type of disease had the best reproducibility. Figure 7 shows the reproducibility results of the eDenseYOLO between the initial and the follow-up CXRs. Figure 8 shows the negative reproducibility results of eDenseYOLO between the initial and follow-up CXRs. Although the reproducibility of PN and PLE was better than the other disease types, they included negative results ((c) and (d)). In addition, the diagnosis of the pleural effusion type showed the best performance, whereas that of the consolidation disease type showed the worst performance, with an average false positives value of 0.6 (Fig. 6). We found that the performance of FROC affected the results ​of each PPA and CPPA. The performance of FROC affected the results of each PPA and CPPA as the recall of CAD in the test set (initial CXRs) and the PPAs and CPPAs have positive correlations (Spearman's rank correlation coefficient, r = 1, *p* = 0.017; r = 1, *p* = 0.017, respectively) in Fig. 9.

## Discussion

Compared to our study, previous studies^10–14^ used statistical analyses for assessing the sensitivity, specificity, accuracy, AUC, and FROC. While these statistical analyses are important for developing CAD systems, we speculated whether the algorithms had a performance for paired CXRs, obtained from the same patient within a short-term interval and whether they could provide a confirmed diagnosis by thoracic radiologists. Using this strategy, we investigated the reproducibility of the CAD system based on deep learning.

Five diseases—including nodules, consolidation, interstitial opacity, pleural effusion, and pneumothorax—were selected as these diseases are important and could be easily confirmed using CT images. There have been many previous studies on CAD developed in response to N^5–8,18^, C, IO^16^, PLE, and PN^12,21–24^. Since CXRs are used as a screening tool in actual clinical settings, the ground truth in this study was generated and confirmed using referral CT images and consensus of two radiologists.

Our results indicate that the reproducibility of eDenseYOLO varied among the five disease patterns. Among the results of the five disease patterns, the PPA and CPPA of the IO and PLE types demonstrated a 100% reproducibility with an average false positives value of 0.6. Although the number of paired CXRS in the IO and PLE types was limited, the reproducibility of these pulmonary lesions was enough to detect the same disease in all paired CXRs except for one case. The PPA and CPPA of the C type pattern were insufficient at 84.21% and 73.21%, respectively. This result included the lack of detection of the C type disease pattern in Fig. 6 and the confirmed diagnosis of the N type disease pattern in the paired CXRs.

The average values of PPA and CPPA for five disease patterns were 93.45% and 88.30%, respectively. Accordingly, this CAD algorithm showed sufficient reproducibility (all values of PPA and CPPA were > 88%). Specifically, we found that the higher values of FROC for our CAD were favorable for better outcomes of PPA and CPPA for each of the diseases. To improve the reproducibility of CAD, it is necessary to increase the FROC value for CAD. We also need to evaluate the reproducibility of CAD with other deep learning algorithms and lesions.

Our study has several limitations. When the radiologists read the CXRs, they reviewed each patient’s follow-up images and evaluated for the presence or absence of the disease. We did not fully consider the actual medical diagnosis to investigate the reproducibility of CAD. Furthermore, the present CAD algorithm was trained without disease progression as the follow-up CXRs were not used for training^15^. Moreover, data on the comparisons of CXRs by human observers were not included. Due to a lack of current GPU memory, all the CXRs were resampled into 1,000 by 1,000 pixels, which could have decreased the clinical validity of classification and detection. Although the registration of the initial and follow-up CXRs is important for evaluating the reproducibility of CAD, registration for co-location of the bounding boxes on both CXR images could be very difficult due to different breath-hold levels, pose, and disease progression. For more accurately evaluating the reproducibility, a proper registration method to evaluate the co-location of the predicted bounding boxes in both initial and follow-up CXRs is required.

In the future, we need to train the CAD algorithm with the follow-up CXRs to enhance its reproducibility and application in clinical settings. Therefore, the reproducibility of CAD could be complemented with methods such as content-based image retrieval (CBIR). Some follow-up CXRs were of a different quality than the initial CXRs, which could lead to false positives or false negatives. As the follow-up CXRs of patients were conducted mainly in emergent situations, follow-up CXRs did not perfectly replicate the initial CXRs. Some were of lower quality due to the motion artifacts produced in emergent situations and the use of different imaging protocols and machines. We aim to apply various augmentation methods to CXRs, including geometric (B-spline transformations, rotate, shift, and zoom), rather than image registration with initial and follow-up CXRs, to improve the reproducibility of CAD. In addition, we aim to apply dedicated registration of initial and follow-up CXRs to evaluate the predicted bounding boxes without various augmentation. Lastly, after collecting more initial and follow-up CXR datasets, including various disease patterns, that have been independently confirmed by expert radiologists in our institutions and additional centers, we will develop algorithms that can reproducibly diagnose the disease from the paired CXR datasets obtained within a short-term interval, thereby improving the performance of the CAD algorithm. Above all, diagnostic results from human operators should be compared with those obtained by deep learning algorithms through a reading test.

In conclusion, CAD systems require reproducibility for their utilization as imaging biomarkers in various clinical settings. Our empirical evaluation of the reproducibility of diagnosis by CAD can be extended to the development of CAD algorithms based on deep learning.

## Abbreviations

AUC: Areas under the curve
CAD: Computer-aided detection
CNN: Convolutional neural network
CPPA: Chamberlain’s percent positive agreement
CXR: Chest radiograph
FROC: Free-response ROC curve
PACS: Picture archiving and communication system
PPA: Percent positive agreement
ROI: Region of interest
YOLO: You only look once
